# Human Umbilical Cord Mesenchymal Stem Cells Infected with Adenovirus Expressing *HGF* Promote Regeneration of Damaged Neuron Cells in a Parkinson's Disease Model

**DOI:** 10.1155/2014/909657

**Published:** 2014-09-03

**Authors:** Xin-Shan Liu, Jin-Feng Li, Shan-Shan Wang, Yu-Tong Wang, Yu-Zhen Zhang, Hong-Lei Yin, Shuang Geng, Hui-Cui Gong, Bing Han, Yun-Liang Wang

**Affiliations:** ^1^Weifang Medical University, 7166 Baotong Road, Weifang 261053, China; ^2^The Neurology Department, The 148th Hospital, 20 Zhanbei Road, Zibo 255300, China; ^3^Medical College of Henan University, 357 Jinming Road, Kaifeng 475001, China

## Abstract

Parkinson's disease (PD) is a neurodegenerative movement disorder that is characterized by the progressive degeneration of the dopaminergic (DA) pathway. Mesenchymal stem cells derived from human umbilical cord (hUC-MSCs) have great potential for developing a therapeutic agent as such. HGF is a multifunctional mediator originally identified in hepatocytes and has recently been reported to possess various neuroprotective properties. This study was designed to investigate the protective effect of hUC-MSCs infected by an adenovirus carrying the* HGF* gene on the PD cell model induced by MPP+ on human bone marrow neuroblastoma cells. Our results provide evidence that the cultural supernatant from hUC-MSCs expressing HGF could promote regeneration of damaged PD cells at higher efficacy than the supernatant from hUC-MSCs alone. And intracellular free Ca^2+^ obviously decreased after treatment with cultural supernatant from hUC-MSCs expressing HGF, while the expression of CaBP-D28k, an intracellular calcium binding protein, increased. Therefore our study clearly demonstrated that cultural supernatant of MSC overexpressing* HGF* was capable of eliciting regeneration of damaged PD model cells. This effect was probably achieved through the regulation of intracellular Ca^2+^ levels by modulating of CaBP-D28k expression.

## 1. Introduction

Parkinson's disease (PD) was first described by British physician James Parkinson in 1817, which is a progressive degenerative disease of the central nervous system of the elderly. The clinical symptoms include tremor, muscle rigidity, bradykinesia, and postural instability, which seriously affect the quality of life. The main pathological changes are believed to be the degeneration of dopaminergic neurons in the substantia nigra pars compacta (SNpc) and their terminals in the striatum [1, 2]. Current treatment for PD relies on medicine, such as levodopa, that alleviates early symptoms but failed to prevent disease progression. In recent years, cell and gene therapies have gained traction in the treatment of PD with the focus on the regeneration of dopamine (DA) producing neurons [3, 4]. Mesenchymal stem cells are multipotent that can be differentiated into various cells of mesodermal lineage. They can be obtained from several sources including bone marrow, adipose tissue, placenta, umbilical cord, and cord blood. Human umbilical cord mesenchymal stem cells (hUC-MSC) due to its availability through noninvasive procedure have demonstrated advantages that become one of the top choices for repairing the damaged neurons [5]. Hepatocyte growth factor (HGF) is a multifunctional growth factor produced by stromal cells. It activates the signal transduction cascade through tyrosine phosphorylating its protooncogenic c-Met receptor. Although it was discovered originally as a growth factor for hepatocytes, it has been demonstrated to be involved in differentiation, proliferation, and regeneration of variety of cells [6–8]. Expression of HGF is found in human brain tissue and is believed to be a survival factor for motor and sensory neurons [9–11]. Salehi and Rajaei reported that HGF could be involved in the pathogenesis of Parkinson's disease [12]. One of the sources for HGF is MSC itself, and it has been shown that HGF signaling plays a critical role during organogenesis [13].

Our previous study has shown that hUC-MSC being infected by an adenovirus carrying the* HGF* gene (Ad-*HGF*) can express dopaminergic neuron specific marker tyrosine hydroxylase and dopamine transporter; in addition, the dopamine levels in the cultural medium of these cells increase significantly [14]. Our finding indicated that hUC-MSC when overexpressing HGF has the differentiation potential for dopaminergic neuron. We hypothesized that the supernatant of hUC-MSC-HGF may contain important factors for neuron cell differentiation and damage repair. To verify that, we designed the present study to treat PD model cells with cell culture supernatant of hUC-MSC overexpressing HGF, and the recovery of cell viability was observed and mechanisms were investigated.

## 2. Materials and Methods

### 2.1. Reagents, Antibodies, and the Expression Vectors

Adenovirus expressing green fluorescent protein gene (Ad-*GFP*) was provided by Beckman Medical Instruments, USA. Recombinant adenovirus carrying* HGF* gene (Ad-*HGF*) was constructed in our lab. Umbilical cord tissue was obtained from the Gynecology Department of the 148 Hospital. All donors have signed informed consent and the study was approved by the ethics committee of the 148 Hospital. MPTP, CaBP-D28k antibody, and Fluo-3-AM were purchased from Sigma (USA).

### 2.2. hUC-MSC Preparation

Isolation and verification of hUC-MSC were carried out based on previously described protocols, and determination on Ad-*HGF* optimal transfection efficiency was performed based on protocols described previously [14].

### 2.3. Preparation of Conditioned Medium

The hUC-MSC cells were infected by Ad-*HGF* at 200 MOI for 48 hours, and the supernatant (CM-HGF) was centrifuged using ultrafiltration tubes at 3000 r/min at 4°C for 1.5 h. The centrifugation was repeated three times until the original culture supernatant was concentrated for 7.5 folds and subsequently was stored at −80°C. Supernatant from hUC-MSC cells culture medium without adenovirus infection (CM-MSC) was also centrifuged in the same manner.

### 2.4. Analysis of HGF Protein Level by ELISA

The hUC-MSCs were transfected with Ad-*HGF* at a MOI of 200 in serum-free F12 for 2 h. The supernatants were harvested at different time-points after transduction (24 h, 48 h, and 72 h). Concentrations of immunoreactive HGF in the supernatant were measured by enzyme linked immunosorbent assay (ELISA). ELISA plates (R&D system) were coated overnight at room temperature with 100 *μ*L of a 12 *μ*g/mL solution of affinity purified anti-HGF diluted in 1X antibody coating buffer. Following 2 washes with 1X wash buffer, the plate was blocked with 300 *μ*L of 1X General Blocker Buffer for 3–6 h at room temperature. Blocking solution was removed, and the plate firmly was tapped on absorbent paper to remove excess liquid and was used immediately. Ninety-five *μ*L of general assay dilutant was added to each well, followed by 5 *μ*L of standard blank (purified HGF) or serum samples. The plate was then sealed and incubated overnight at 4°C. Following 5 washes with 1X washing buffer, 100 *μ*L of HRP-conjugated secondary antibody was added to each well and the plate was incubated again for 1 h at room temperature. The plate was washed 5 additional times. After complete removal of excess solution, 100 *μ*L of TMB substrate was added to each well. Following a 15-minute incubation period at room temperature, 100 *μ*L of stop solution was added and the absorbance at 450 nm was read using a plate reader (Bio-Rad). Concentrations of HGF in the samples were calculated relative to the exponential standard curve obtained from the standard included in each assay.

### 2.5. Preparation of PD Cell Model

SH-SY5Y cells in logarithmic growth phase were incubated in 96 well plates at a density of 5 × 10^4^/mL and 100 *μ*L/well for 12 h and then treated with MPP+ of various concentrations for 24 h. Cell viability was assessed by adding 10 *μ*L of CCK-8 (Dojindo Molecular Technologies) to the culture and continuing incubation for 2 h. Cell viability was measured by spectrometry with 450 nm wavelength. The concentration of MPP+ for desired cell damage was determined for PD cell model based on cell viability.

### 2.6. SH-SY5Y Cell Proliferation Measurement

SH-SY5Y cells are divided into 4 groups: normal cells (control group), cells treated with MPP+ (model group), PD model treated with CM-MSC for 24 or 48 h (CM-MSC group), and PD model treated with CM-HGF for 24 or 48 h (CM-HGF group). Cell viability was assessed by CCK-8 assay as described above.

### 2.7. Observation on Ca^2+^ Changes in SH-SY5Y Cells by Confocal Microscope

SH-SY5Y cells were washed 3 times with PBS after various treatments and incubated with Fluo-3-AM (5 *μ*M) at 37°C or room temperature for 0.5 to 1 h. Cells were rinsed 2~3 times and observed under confocal microscope. Cell images were analyzed by LaserSharp 2000 image analysis software.

### 2.8. Western Blot

Cells were collected and lysed after various treatments indicated. The protein concentration was determined using the Bio-Rad protein assay kit (Bio-Rad, Hercules, CA, USA). The samples were separated by 12% SDS-PAGE and transferred to PVDF membranes. CaBP-D28k expression was detected with rabbit anti-CaBP-D28k antibody (Sigma) and subsequent HRP-conjugated secondary antibody. Image was analyzed for densitometry with BIO-RAD Quantity One software. The blots were probed with an anti-*β*-actin antibody (Sigma) for loading quantity.

### 2.9. Statistical Analysis

Statistical analysis was performed using SPSS10.0 software. One-way ANOVA was used for comparison between two groups. All data were presented as mean ± standard deviation (*x* ± *s*), and *P* < 0.05 was considered statistically significant.

## 3. Results

### 3.1. hUC-MSC Isolation

We have previously shown that hUC-MSC can be successfully isolated from human umbilical cord, which expresses CD29, CD44, and CD105, the known surface markers for mesenchymal stem cells, but not hematopoietic stem cell marker CD45 or epithelia cell marker CD31 [14]. The maximum infection efficiency can be achieved when the hUC-MSC cells were infected by adenovirus at the m.o.i. of 200, as shown by flow cytometry and fluorescence microscopy (data not shown).

### 3.2. The Concentration of HGF Increased in hUC-MSCs Supernatant after Transduction with Ad-HGF

**Figure 1:**
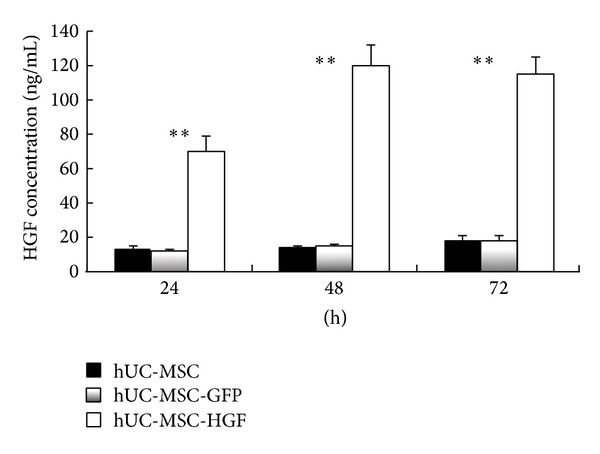
The concentration of HGF in cells supernatant was detected by ELISA. After the hUC-MSCs were transduced with Ad-HGF, HGF gradually accumulated in hUC-MSCs supernatant, peaking at about 120 ng/mL at 48 h. Levels remained stable at about 15 ng/mL in the Ad-GFP and blank control group. Indeed, statistically significant differences emerged between the control and Ad-HGF groups upon analysis (**P < 0.01).

As shown in Figure 1, HGF accumulated to about 75 ng/mL in the supernatant at 24 h in Ad-HGF groups. It gradually increased, peaking at 120 ng/mL at 48 d. At 72 d, concentration of HGF declined slightly to approximately 115 ng/mL. In blank control and Ad-GFP groups, HGF concentration remained stable at about 15 ng/mL (Figure 1). It is clear that HGF protein level obviously increased after infection with Ad-HGF than hUC-MSC cells alone at different time-point (**P < 0.01).

### 3.3. Establishment of PD Model with MPP+ Treated SH-SY5Y Cells

**Figure 2:**
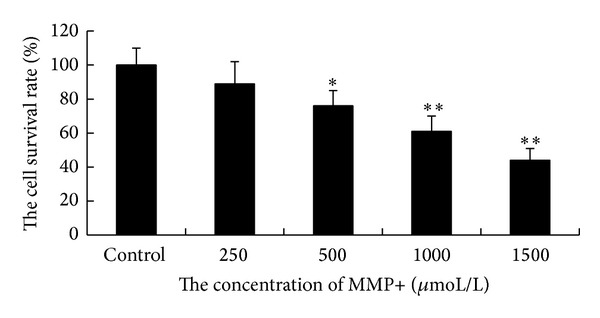
Cell viability of SH-SY5Y after being treated with various concentrations of MMP+ as assessed by CCK-8 assay. The results show that, with the increasing concentration of MMP+, SH-SY5Y cell viability decreased significantly at 500, 1000, and 1500 *μ*M MMP+ compared to the control group (*P < 0.05, **P < 0.01).

Human neuroblastoma cell line SH-SY5Y is one of the widely used cell lines for studying the neurodegeneration and neurotoxicity related to PD. It has been shown that undifferentiated SH-SY5Y cells are susceptible to neurotoxin such as MMP+. SH-SY5Y cells were treated with various concentrations of MMP+ for 24 h. CCK-8 assay was used to assess the survival of the cells after treatment. A decreased cell viability was seen with the increase of the MMP+ concentration. Cell survival was about 90% at 250 *μ*mol/L of MMP+ and dropped to 73.09%, 60.0%, and 50.0% at the concentrations of 500, 1000, and 1500 *μ*M MPP+, respectively. Significant differences were found in the later three groups compared with the control group (Figure 2, *P* < 0.05). Treatment of MMP+ at a concentration of 1000 *μ*M was chosen for PD model.

### 3.4. Protection on Damages of PD Model by CM-HGF and CM-MSC

**Figure 3:**
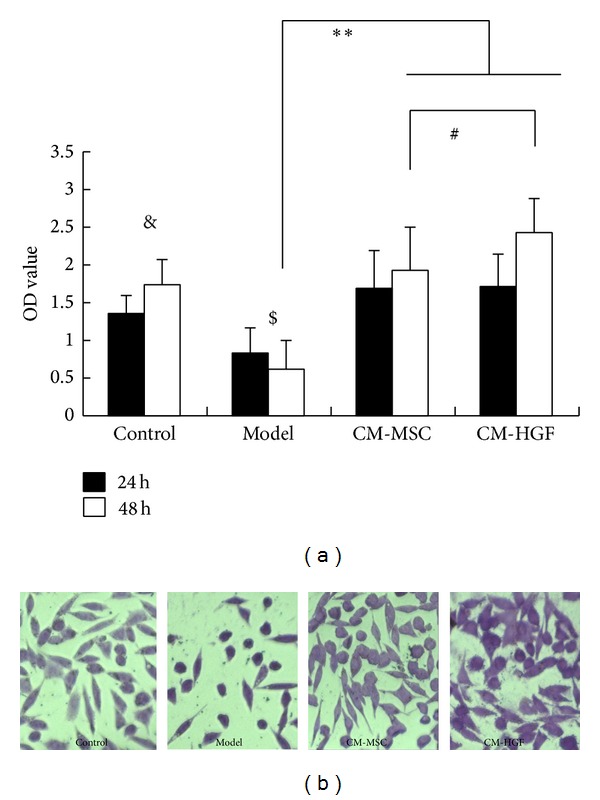
(a) Cell viability of SH-SY5Y underwent various treatments. Control: no-treatment; model: treated with MMP+ at 1000 *μ*M; CM-MSC: treated with MMP+ at 1000 *μ*M and then incubated with cultural supernatant of hUC-MSC; CM-HGF: treated with MMP+ at 1000 *μ*M and then incubated with cultural supernatant of hUC-MSC infected with Ad-*HGF*. Viability in PD model cells was significantly decreased compared to control (^$^ *P* < 0.01). After being treated with either CM-HGF or CM-MSC for 48 h, cell viability was significantly increased compared to the PD model (**P < 0.01) and compared with the normal control group (^&^ *P* < 0.05). Significant difference between CM-MSC and CM-HGF treatment was seen after 48 h treatment (^#^ *P* < 0.05). (b) Microscopic images of cells with various treatments. The SH-SY5Y cells under each treatment were stained with crystal violet and observed under an inverted microscope.

SH-SY5Y cells treated with MMP+ at 1000 *μ*M for 24 h were used as the established PD model. These cells were incubated with CM-HGF or CM-MSC, and CCK-8 assay was employed to study the viability of the cells. As shown in Figure 3(a), both CM-HGF and CM-MSC treatments were able to regenerate the damaged SH-SY5Y cells. At 48 hours after treatment, the proliferative effect from CM-HGF was more significant than that from CM-MSC (*P* < 0.05). Cell viability of PD model cells treated with CM-HGF was significantly higher compared to that of CM-MSC treated or untreated normal cells. Viability of PD model cells treated with CM-MSC as measured by O.D. 450 showed no significant difference compared to the normal control group at 48 h culture (*P* > 0.05), while the OD 450 value of the CM-HGF treated group was significantly higher than the normal control group (*P* < 0.05, Figure 3(a)). These results suggested that CM-HGF and CM-MSC could promote regeneration of SH-SY5Y. Under the microscope, while the control cells were showed in good condition with clear edge, most of the PD model cells (MMP+ treated for 72 h) appeared to have nuclear condensation and drastically shrunken cell bodies. However, these morphologies were significant improved after these cells were treated with CM-HGF and CM-MSC (Figure 3(b)).

### 3.5. Intracellular Ca^2+^ after Being Treated with CM-HGF and CM-MSC

**Figure 4:**
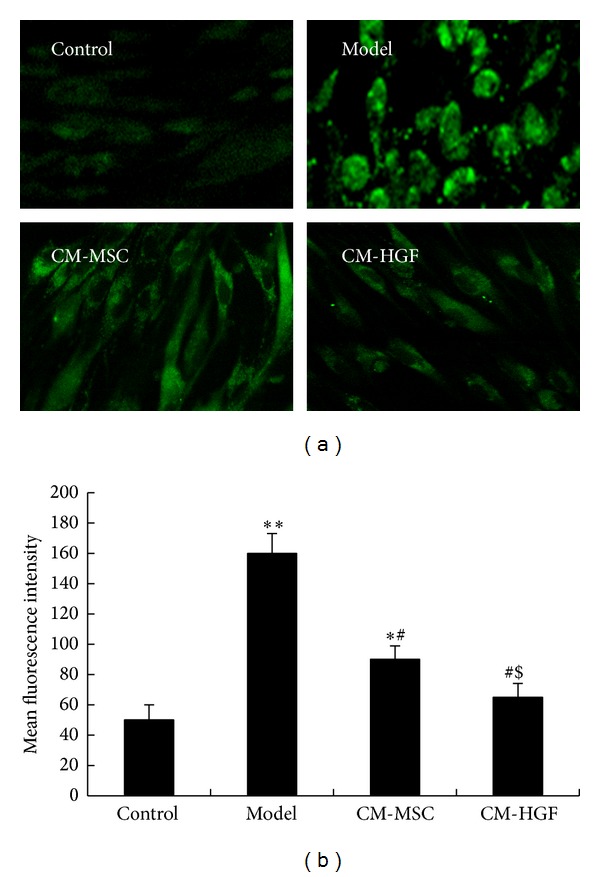
(a) Confocal microscope image of cells underwent various treatments. Fluo-3-AM was added to cells after incubation with CM-MSC or CM-HGF, and fluorescence was observed with LSCM. (b) Fluorescence intensity analysis with LaserSharp 2000 software. *P < 0.05, between CM-MSC and control; **P < 0.01, between PD model and control; ^#^ *P* < 0.01, between CM-HGF or CM-MSC and PD model; ^$^ *P* < 0.05, between CM-MSC and CM-HGF.

Fluo-3-AM can be used as a calcium indicator due to its property of marked increased fluorescence intensity when it is bound with Ca^2+^. It has low affinity for Ca^2+^ and can be readily dissociated. Therefore, it is ideal for measuring rapid and minimal changes of intracellular Ca^2+^. LSCM was used to study the intracellular Ca^2+^ in these cells. Compared with the normal control cells, PD model cells showed enhanced intracellular fluorescence upon Fluo-3-AM staining, indicating an increase of intracellular free Ca^2+^. Intracellular fluorescence intensity weakened after these cells were treated with CM-HGF or CM-MSC, indicating decreased intracellular free Ca^2+^ (Figure 4(a)). Cell fluorescence intensity was quantified with LaserSharp 2000 software by quantifying randomly picked 10 cells in each optical area (Figure 4(b)). The fluorescence intensity in cells of the PD model was significantly higher than that of the normal control cells (*P < 0.05), whereas the fluorescence intensity in cells of the PD model treated with CM-MSC, though lower than that of the untreated PD model cells, was still significantly higher than that of the normal cells (**P < 0.01). The fluorescence intensity of PD model treated with CM-HGF was higher than that of the normal control, but there is no significant difference between them (*P* > 0.05). Comparing the intracellular fluorescence intensity between PD model cells and PD model cells treated with either CM-HGF or CM-MSC, it was found that both treatments significantly decreased the intracellular free Ca^2+^ levels (^#^ *P* < 0.01). In addition, CM-HGF treatment showed a better efficacy in reducing the intracellular free Ca^2+^ levels than CM-MSC treatment, as indicated by the lower fluorescence intensity in CM-HGF treated cells than in the CM-MSC treated cells (^$^ *P* < 0.05, Figure 4(b)).

### 3.6. Expression of CaBP-D28k in CM-MSC and CM-HGF Treated Cells

**Figure 5:**
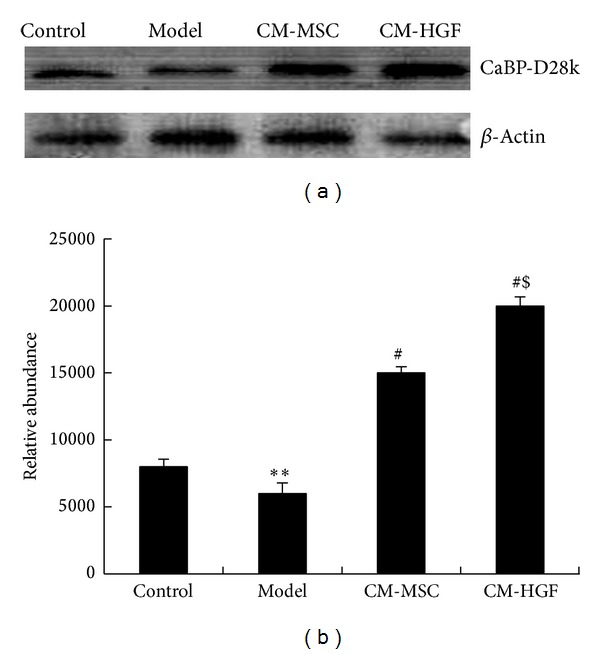
(a) Western blot results of total cell lysates for CaBP-D28k expression after various treatments. (b) Densitometry analysis with Quantity One software. *β*-actin was used as a loading quantity reference on which the expression levels of CaBP-D28k were normalized. **P < 0.01, between model cells and control; ^#^ *P* < 0.05, between CM-HGF or CM-MSC and model cells; ^$^ *P* < 0.05, between CM-HGF and CM-MSC.

Calbindin CaBP-D28k is a high affinity calcium-binding protein that plays an important role in calcium homeostasis. CaBP-D28k has been indicated to confer protection to SNC dopaminergic neurons against certain pathological process related to PD. The expression of CaBP-D28k was assessed by Western blot analysis in the PD cell model that underwent various treatments. As shown Figure 5, the expression of CaBP-D28k in SH-SY5Y cells treated with MPP+ (PD model cell) was significantly decreased compared to the normal cells. However, when these cells were treated with CM-MSC or CM-HGF, the expression of CaBP-D28k was elevated. Densitometry analysis showed that the differences between the normal control and PD model cells were statistically significant (**P < 0.01). Both CM-HGF and CM-MSC treatments significantly upregulated CaBP-D28k expression (^#^ *P* < 0.05); however, the efficacy of CM-HGF was shown to be significantly more potent than that of CM-MSC (^$^ *P* < 0.05).

## 4. Discussion

MSCs are multipotent stem cells that can be easily obtained and expanded without getting involved with ethical issues. MSCs are of low immunogenicity, able to pass through the blood-brain barrier after intravenous transplantation, and have long survival time after transplantation. hUC-MSCs are particular attractive because they can be procured through noninvasive procedure and have abundant sources [5, 15]. MSCs have been shown to migrate to the site of brain injury, and safety of MSCs transplantation into brains has been demonstrated; thus, they are attractive therapeutic options for neurodegenerative disorders [16–19].

The major pathogenesis of PD is its loss of DA neurons, thus making it a good candidate for cell therapy. A lot of attentions have been given to use DA neurons differentiated from stem cells of various sources to replace the degenerated DA neurons [20]. However, studies have been shown that stem cells are also capable of protecting or stimulating the regeneration of damaged DA neurons in host [21, 22].

*HGF* is widely expressed in the nervous system, although its association with PD was still not clear. Salehi and Rajaei detected higher HGF concentrations in cerebrospinal fluid of PD patients than that of the normal population [12]. Lan et al. found that HGF can mediate proliferation and migration of primary dopaminergic nerve progenitor cells separated from the placenta [23]. A group in Japan injected plasmid carrying* HGF* gene into PD rat model and found significant reduction of symptoms, suggesting that overexpression of* HGF* can prevent death of dopaminergic neurons in Parkinson rats [24].

Exogenous HGF protein has a very short half-life* in vivo*, and more critical, HGF is a macromolecular protein and cannot pass through the blood-brain barrier [8, 12]. Meanwhile, mesenchymal stem cells are appropriate cell carriers for exogenous genes [25]. Therefore, we decided to explore the idea of the combination of HGF and hUC-MSCs as a treatment of PD, by introducing* HGF* gene into hUC-MSCs to establish HGF producing MSCs. In our study, the supernatant from hUC-MSCs infected with Ad-*HGF* was more potent in inducing PD model cell regeneration than the supernatant from hUC-MSCs noninfected cells. Our data suggested that combination of HGF and hUC-MSCs had advantage over hUC-MSCs alone.

Damage to DA neurons can be triggered by toxin exposure, increased oxidative stress, and mitochondrial dysfunction, protein aggregation, and inflammation [26]. Calcium is important to normal cell function. Intracellular calcium regulation is closely related to mitochondrial function and oxidative stress, and there is increasing evidence that disruption of intracellular calcium homeostasis is crucial to the pathogenesis of PD [27]. CaBP-D28k is an intracellular calcium binding protein that is expressed in many neurons. It has been shown that neuron expressing CaBP-D28k is less susceptible to the damage [28]. CaBP-D28k is shown to activate Ca^2+^/Mg^2+^-ATPase, preventing excessive Ca^2+^ accumulation in the brain, thus, playing a role in Ca^2+^ transport and maintaining calcium homeostasis in the neurons [29, 30]. Recent studies also show that the levels of CaBP-D28k protein in neurons not only represent the Ca^2+^ change but are also closely related to the integrity of the structure and function [31]. Our present study demonstrated that intracellular free Ca^2+^ levels were increased in the PD model cells, and the reductions of the Ca^2+^ levels were correlated to the recovery of cell viability elucidated by the treatment of CM-HGF or CM-MSC. Moreover, CaBP-D28k expression levels in SH-SY5Y PD model cells were reversely correlated with the intracellular Ca^2+^ levels. Expression of CaBP-D28k was decreased in PB model cells but increased after these cells were treated with CM-HGF and CM-MSC. Similar to its effect on PD model cell regeneration, CM-HGF was more potent than CM-MSC in reducing intracellular Ca^2+^ levels and promoting CaBP-D28k expression. Our study clearly demonstrated that cultural supernatant of MSC overexpressing* HGF* was capable of eliciting regeneration of damaged PD model cells. This effect was probably achieved through the regulation of intracellular Ca^2+^ levels by modulating of CaBP-D28k expression. Further studies are needed to understand the active components in the cultural supernatant and the signaling cascade involved.
